# Structure and Nanomechanics of Model Membranes by Atomic Force Microscopy and Spectroscopy: Insights into the Role of Cholesterol and Sphingolipids

**DOI:** 10.3390/membranes6040058

**Published:** 2016-12-19

**Authors:** Berta Gumí-Audenis, Luca Costa, Francesco Carlá, Fabio Comin, Fausto Sanz, Marina I. Giannotti

**Affiliations:** 1 Nanoprobes and Nanoswitches group, Institute for Bioengineering of Catalunya (IBEC), Barcelona 08028, Spain; bgumi@ibecbarcelona.eu (B.G.-A.); fsanz@ub.edu (F.S.); 2 Physical Chemistry Department, Universitat de Barcelona, Barcelona 08028, Spain; 3 European Synchrotron Radiation Facility (ESRF), Grenoble 38043, France; francesco.carla@esrf.fr (F.C.); comin@esrf.fr (F.C.); 4 Networking Biomedical Research Center on Bioengineering, Biomaterials and Nanomedicine (CIBER-BBN), Madrid 28028, Spain; 5 Structure and Dynamics of Nucleoproteic and Membrane Assemblies, Centre de Biochimie Structurale (CBS), Montpellier 34090, France; costa@cbs.cnrs.fr

**Keywords:** atomic force microscopy, force spectroscopy, lipid membranes, supported lipid bilayers, nanomechanics, cholesterol, sphingolipids, membrane structure, XR-AFM combination

## Abstract

Biological membranes mediate several biological processes that are directly associated with their physical properties but sometimes difficult to evaluate. Supported lipid bilayers (SLBs) are model systems widely used to characterize the structure of biological membranes. Cholesterol (Chol) plays an essential role in the modulation of membrane physical properties. It directly influences the order and mechanical stability of the lipid bilayers, and it is known to laterally segregate in rafts in the outer leaflet of the membrane together with sphingolipids (SLs). Atomic force microscope (AFM) is a powerful tool as it is capable to sense and apply forces with high accuracy, with distance and force resolution at the nanoscale, and in a controlled environment. AFM-based force spectroscopy (AFM-FS) has become a crucial technique to study the nanomechanical stability of SLBs by controlling the liquid media and the temperature variations. In this contribution, we review recent AFM and AFM-FS studies on the effect of Chol on the morphology and mechanical properties of model SLBs, including complex bilayers containing SLs. We also introduce a promising combination of AFM and X-ray (XR) techniques that allows for in situ characterization of dynamic processes, providing structural, morphological, and nanomechanical information.

## 1. Introduction

Biological membranes are self-sealing boundaries, confining the permeability barriers of cells and organelles and yielding the means to compartmentalize functions. Apart from being crucial for the cell structure, they provide a support matrix for all the proteins inserted in the cell. Biological membranes mediate several biological processes—cell recognition and signaling, ion transference, adhesion, and fusion—directly affecting their physical properties, which are sometimes difficult to evaluate. Lateral and transverse forces within the membrane are significant and change rapidly as the membrane is bent or stretched and as new constituents are added, removed, or chemically modified. Differences in structure between the two leaflets and between different areas of the bilayer can associate with membrane deformation to alter the activities of membrane-binding proteins [1,2].

Lipids are the main component of biological membranes besides proteins and carbohydrates. Lipids show a well-defined organization and many cellular membranes are asymmetric. The internal leaflet of plasma membranes is typically composed of charged phosphatidylserines, phosphatidylethanolamines, and a smaller number of phosphatidylcholines (PCs), while the outer leaflet is mostly composed of PCs and sphingolipids (SLs), including glycolipids. Cholesterol (Chol), present in both leaflets, is also an important component of the cell membrane, while transmembrane distribution remains debatable [3]. It has been experimentally shown that the membrane is able to laterally segregate its constituents, subcompartmentalizing them into small domains (10–200 nm) known as rafts [4,5]. The so-called rafts are fluctuating nanoscale assemblies of lipids, enriched with Chol, SLs, and proteins, that seem to play significant biological roles in membrane signaling and trafficking [4,6].

Chol is a fundamental component of eukaryotic cells and can reach concentrations up to 50 mol % of the overall lipid contained in cell plasma membranes. Certainly, Chol plays an essential role in modulating membrane physical properties, being highly important in the function and evolution of the biological membrane [2,7]. It regulates membrane fluidity, controls the lipid organization and phase behavior, and increases the mechanical stability of the membrane [8,9,10]. From a molecular point of view, Chol produces a condensing effect by ordering the fluid phase lipids in the membrane, which leads to an increase in the bilayer thickness and a decrease in its permeability [11,12]. Nevertheless, many studies highlight that the effect of Chol on the lipid bilayers depends on the molecular structure of the neighboring lipids [8], especially on the degree of chain unsaturation [13], the length of the hydrophobic tails [14], and the chemical composition of the headgroup. However, Chol is generally accompanied by SLs in rafts, playing a joint effect on the structural and nanomechanical properties of the lipid bilayer. Thus, it is of great significance to understand the nanomechanical behavior of lipid bilayers and the physical function each membrane component has.

Considering the complex chemical diversity of biological membranes, model bilayer systems are frequently used to study membrane properties and biological processes that occur at the cellular or subcellular level [15,16]. For instance, phospholipid bilayers are very manageable platforms resembling cell membranes: they retain two-dimensional order and lateral mobility and offer excellent environments for the insertion of membrane proteins. Nowadays, a wide range of supported systems have emerged as suitable approaches for biological studies and sensor design [17], like self-assembled monolayer–monolayer systems, polymer-cushioned phospholipid bilayers, or bilayer coated microfluidics, among others. However, supported lipid bilayers (SLBs) or supported planar bilayers (SPBs) facilitate the use of surface analytical techniques. SLBs are ideal platforms to study the lipid lateral interactions, the growth of lipid domains [18], as well as interactions between the lipid membrane and proteins, peptides and drugs [19], cell signaling, etc.

**Figure 1:**
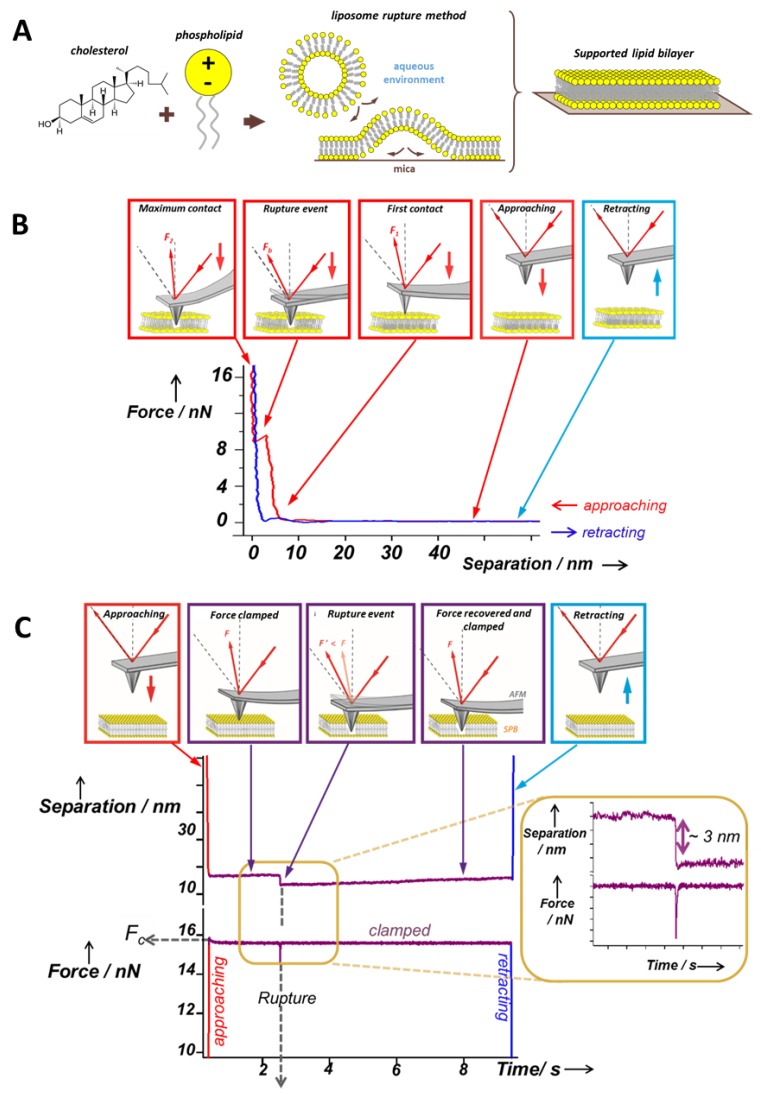
(**A**) Schematic diagram showing the formation of SLBs via the liposome rupture method; (**B**) schematics of the SLB indentation process using AFM-based force spectroscopy (AFM-FS), displaying a force–separation typical curve, showing the discontinuity in the approach curve when the bilayer is punctured. The different steps in the scheme and the corresponding part of the force curve are linked by arrows. (**C**) Schematics of the SLB indentation process under constant force: AFM-based force clamp (AFM-FC), displaying separation-time and force-time typical curves, showing the bilayer rupture event. The different steps in the scheme and the corresponding part of the curves are linked by arrows. Adapted with permission from ref. [24]. Copyright 2012 American Chemical Society.

Among the several methods to obtain SLBs [15], the most widely used are the Langmuir–Blodgett (LB) technique [20] to prepare mono and bilayers, the hydration of spin-coated films [21], and the liposome rupture or fusion method, to prepare bilayers. The liposome rupture method, the most popular and simple, consists of the fusion of small unilamellar vesicles (SUVs) from a suspension as soon as they come in contact with a flat substrate (Figure 1A). Then, the SUVs will start fusing them, deforming, flattening, and finally rupturing to form a continuous SLB [15]. In any case, the mechanism to obtain bilayers from SUVs is not fully understood. Variables concerning the lipid vesicles (composition, concentration, and size), the physicochemical environment (pH, temperature, and ionic strength), and the surface (roughness and charge density) have been reported to highly influence the final SLB structure [22]. Hence, it is important to consider the substrate when interpreting the results from a characterization of SLBs. Mica is the most common material used as a substrate, since it is easy to cleave and get a clean surface, atomically flat and hydrophilic. Apart from mica, other alternative substrates can be used [23], e.g., borosilicate glass, silicon oxide, or even gold surfaces.

Several reports demonstrate the wide variety of techniques used to study supported and non-supported lipid membranes, including fluorescence microscopy [25], fluorescence recovering after photobleaching (FRAP) [26], Brewster angle microscopy (BAM) [27], ellipsometry, X-ray [28,29,30], and neutron [31,32] techniques, among others. Focusing on investigating the physical properties of lipid bilayers, micropipette aspiration has proven to be remarkable in the determination of elastic moduli of the membrane, even though this technique can only be applied to giant vesicles [33]. Thanks to the possibility of working in a controlled environment and with distance and force resolution at the nanoscale, atomic force microscopy (AFM) is now a well-established technique for both imaging the morphology and probing the local physical and mechanical properties of SLBs by means of force spectroscopy modes [10,16,34,35,36].

Although several articles review the use of AFM to study model membranes mechanics, in this contribution we review the AFM-based approach to evaluate the structure and nanomechanics of model membranes, focusing on recent studies on the effect of Chol on model SLBs under temperature variations. We also discuss AFM investigations on more complex bilayers containing SLs, which together with Chol are key structural molecules of the lipid membrane. Furthermore, we introduce the promising combination of AFM and X-ray (XR) techniques, allowing for in situ characterization of dynamic processes, providing at once structural, morphological, and nanomechanical information. We present the first results on simple model membranes using this combination and perspectives for its future application to complex SLBs.

## 2. AFM: Topographical and Mechanical Characterization of SLBs

**Figure 2:**
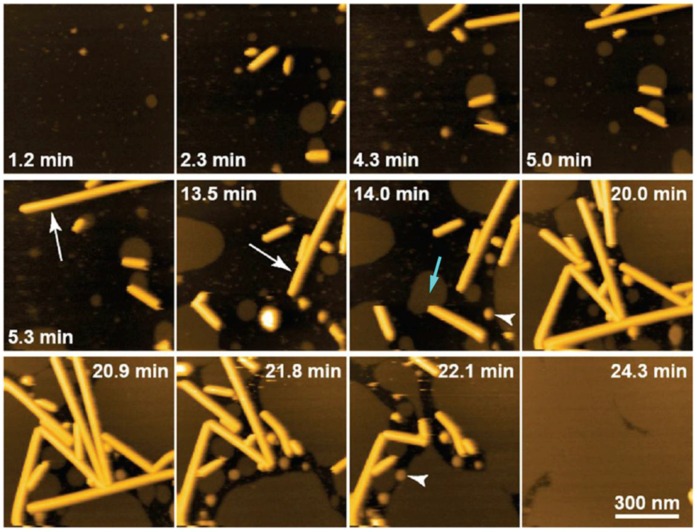
HS-AFM imaging of the growth of lipid nanotubes of about 20 nm height occurring in the process of SLB formation on mica. The white arrows indicate rapidly growing lipid nanotubes. The light-blue arrow indicates the interaction between an SLB patch and one end of a lipid nanotube. The arrowheads indicate liposomes. Adapted with permission from [41]. Copyright 2014 American Chemical Society.

Since AFM was born in 1986 [37], it has been an essential technique to explore a wide range of samples at the nanoscale. The main advantage of AFM is the possibility of controlling the environmental conditions (medium composition and temperature) while applying and sensing minimal forces (pN to nN range), consequently enabling us to operate in a liquid environment on a large variety of biological samples; from single molecules, i.e., DNA or proteins, to macromolecular assemblies such as SLBs or even whole cells [38]. AFM has become a well-established technique for imaging the lateral organization of lipid membranes that show homogeneous or phase separated SLBs [16,36]. Compared with other techniques, AFM allows for the structure of biological samples to be imaged in real time—with the possible use of high-speed AFM (HS-AFM) [39,40,41]—and with (sub)nanometer resolution [42]. Figure 2 shows an example where HS-AFM is used to track the deposition of small lipid vesicles onto a mica surface during SLB formation, also showing the unexpected phenomenon of lipid nanotube growth [41].

Thanks to the ability of AFM to sense and apply forces with high accuracy, AFM-based force spectroscopy (AFM-FS) has become an excellent tool to study molecular interactions at the single molecule level [43]. Therefore, during recent decades AFM-FS has been a suitable technique to perform nanomechanical studies on a wide range of systems, such as indenting hard materials while the AFM tip is approaching the surface [44] or pulling individual macromolecules—polysaccharides [43,45], proteins [46,47,48], and DNA [49]—during the retraction of the AFM tip from the surface. In the case of lipid bilayers, AFM-FS has become a very valuable approach to probe the mechanical properties at the nanoscale with high spatial and force resolution [9,34,35,50].

**Figure 3:**
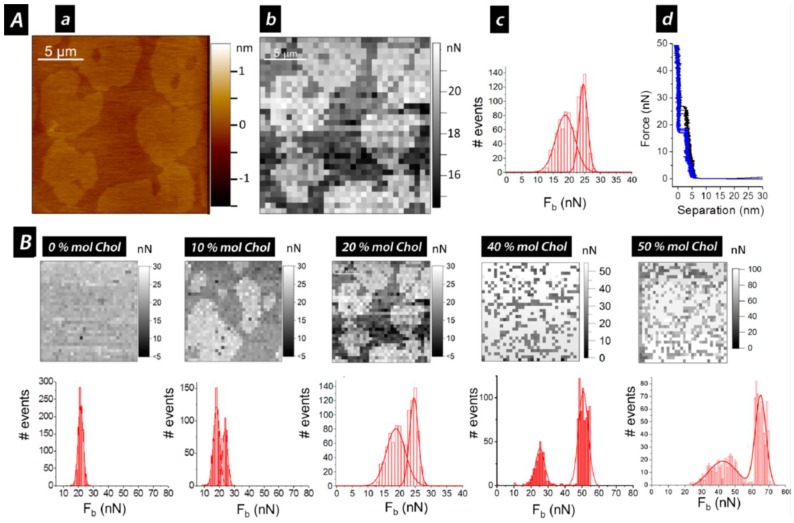
(**A**) DPPC:Chol SPB with 20 mol % Chol in 10 mM HEPES, 20 mM MgCl_2_, and 150 mM NaCl, pH 7.4, at 27 °C: (**a**) AC-mode AFM topographical image; (**b**) the corresponding *F_b_* map; (**c**) the corresponding *F_b_* histogram distribution; (**d**) typical approach force–separation curves of each domain (blue, domains with lower *F_b_* values; black, domains with higher *F_b_* values); (**B**) *F_b_* maps and distributions for DPPC:Chol SPBs in 10 mM HEPES, 20 mM MgCl_2_ and 150 mM NaCl, pH 7.4, at 27 °C, for different Chol content: 0, 10, 20, 40, and 50 mol % Chol. Scan sizes are 10 × 10 μm^2^ for 0 and 10 mol % Chol, and 20 × 20 μm^2^ for 20, 40, and 50 mol % Chol. Adapted with permission from [9]. Copyright 2012 American Chemical Society.

Experimentally, an SLB patch is first located by AFM imaging the sample. Then, the AFM tip away from the surface is approached and retracted at constant velocity. Upon mechanical contact, the cantilever deflection increases and the SLB is elastically compressed by the AFM probe until the tip suddenly breaks and penetrates through the bilayer, coming into direct contact with the substrate (Figure 1B). The penetration of the AFM tip through the bilayer appears as a discontinuity in the approaching force–separation curve (the red curve in Figure 1B). The step observed in the separation correlates with the thickness of the SLB. The vertical force at which this discontinuity happens corresponds to the maximum force the bilayer is able to stand before breaking and is defined as breakthrough force (*F_b_*). *F_b_* usually occurs at several nN and is considered as a direct measurement of the lateral interactions between lipid molecules. Previous reports show that *F_b_* is significantly altered due to variations in the chemical structure of the phospholipid molecules [51,52] and in the physicochemical environment (temperature, pH, or ionic strength) [10,52,53,54]. Therefore, *F_b_* is considered the fingerprint of the mechanical stability of a certain lipid bilayer under specific environmental conditions. In multicomponent SLBs, the *F_b_* value can be directly associated with the membrane composition of homogeneous systems or phase-segregated domains [9,55,56]. Hence, force spectroscopy measurements helps us to better understand the nature of the different phases observed in the AFM topographical images, thanks to what is called a force map. After imaging the selected area, several force–distance curves are created by following a grid in the same scanned region. Extracting the values of the desired mechanical parameters, a force map correlating the topography can be built, as well as the corresponding distribution in order to get the mean values for each variable. For instance, values of *F_b_*, adhesion forces, and height obtained from force–distance curves can be associated with the different gel and liquid domains observed in the topography of phase-segregated SLBs [9], as exemplified in Figure 3A for a DPPC (1,2-dipalmitoyl-*sn*-glycero-3-phosphocholine, 16:0 PC; *T_m_* = 41 °C) bilayer that contains 20 mol % of Chol and is phase segregated in domains of different composition, easily observed in the topographical image (a), and that display different mechanical resistance, as shown in the *F_b_* map (b) and bimodal *F_b_* distribution (c).

The nature of the mechanical rupture of lipid bilayers is based on thermal fluctuations and their destructive action is facilitated and directed by the application of an external force. So far, the penetration of the AFM tip into SLBs has been modeled and widely conceived as a two-state activated process with an associated energy barrier [57,58,59]. In particular, two specific models describing the activation process have been proposed. Firstly, the so-called continuum nucleation model, which takes into account a molecular thin homogeneous film (a two-dimensional fluid layer) between the solid substrate and the solid surface of the AFM tip. The second model, considering the molecular nature of the lipid bilayer, proposes that each molecule in the SLB has specific binding sites corresponding to energetically favorable positions. While the tip is away from the lipid film, these sites are energetically equivalent, whereas as soon as the SLB is pressed by the tip, the energy of the molecules significantly increases, leading them to jump apart and create a hole under the tip. After a critical number of phospholipids have jumped out of the contact area, the tip indents the SLB due to the high pressure of the remaining molecules breaking the bilayer. For this reason, characterization of the energy barriers governing the lipid membranes rupture process is important to gain a better understanding of the extent of the lateral interactions in the bilayer.

Dynamic Force Spectroscopy (DFS) is based on registering the *F_b_* for a bilayer in a defined environment at different constant approaching velocities of the tip to the surface [56,60,61,62]. Taking into account the dependence of *F_b_* on the loading rate, DFS allows for the calculation of the activation energy of the bilayer rupture in the absence of an external force (*E_0_*) [60,62]. However, the location of the energy barrier maximum along the reaction coordinate (*Δx*) cannot be assessed by means of DFS at constant temperature, but requires further investigation of the process at various temperatures [61]. A recent work introduced the use of AFM-based force clamp (AFM-FC), well-established in the study of stepwise unfolding of proteins and other macromolecules at a constant pulling force [63], as a distinct approach to directly characterize the kinetics of the lipid bilayer rupture [24]. Contrarily to conventional AFM-FS measurements, where the tip moves at constant velocity while the force is measured, AFM-FC works by controlling the applied force at a fixed value (*F_c_*) while registering the tip position (separation) in time (Figure 1C). The bilayer rupture is identified as a sudden force drop (and recovery to the clamped force) in the force–time curves and as a step in separation–time curves. This single-step corresponds to the average thickness of the SLB also observed in the force–separation curves for AFM-FS experiments at constant velocity. The time at which the bilayer is ruptured is the time to breakthrough (*t_b_*) and, for each particular *F_c_*, *t_b_* shows an exponential decay distribution that defines the mean lifetime and rate of the rupture process *α*. The dependence between *α* and *F_c_* follows the Arrhenius–Bell expression [64,65] and allows us to calculate both *E_0_* and *Δx*, giving direct information about the kinetics behind the SLB failure process.

AFM coupled to a temperature control system has been found to be a suitable tool to investigate the topographical and mechanical evolution at the nanometer scale of biological processes that are temperature-dependent. It allows for obtaining relevant information about the structural and physical changes of the membrane occurring during the phospholipid phase transitions [9,53,66,67]. Recently, insights on the dynamics of the DMPC (1,2-dimyristoyl-*sn*-glycero-3-phosphocholine, 14:0 PC; *T_m_* = 24 °C) transition from ripple phase to fluid phase reversibly in real time by HS-AFM have also been reported [68]. A second type of ripple phase with larger periodicity has been identified when heating DMPC SLBs from the ripple phase to the fluid phase.

Phase transitions are also evidenced by means of AFM-FS. Temperature has a strong effect on the *F_b_* values of gel-like phospholipid bilayers, like the case of DPPC, whereas less impact is observed for the fluid-like phase, such as DOPC (1,2-dioleoyl-*sn*-glycero-3-phosphocholine, 18:1 (Δ9-Cis) PC; *T_m_* = −17 °C) [9,53,69], allowing us to determine the phase transition following the evolution of *F_b_* when varying the temperature.

## 3. Cholesterol’s Effect on Phosphatidylcholine SLBs

Chol is well known to control the behavior of the physical properties of lipid membranes depending on the molecular structure of the neighboring lipids. X-ray scattering studies in the low angle and wide angle regions have shown that Chol tends to produce a larger effect on lipids with saturated chains compared to the ones containing unsaturations [8,13].

Chol tends to affect the bilayer by condensing the membrane and ordering the lipid molecules, although it depends on the chemical structure of the lipids in the SLB. Chol completely dissolves in fluid-like liquid disordered (*l_d_*) membranes like DOPC and DLPC (1,2-dilauroyl-*sn*-glycero-3-phosphocholine, 12:0 PC; *T_m_* = −2 °C). Both AFM and AFM-FS show that pure DOPC and DLPC SLBs are homogeneous and display mean *F_b_* values of 10 nN and 2 nN, respectively, at room temperature [9,62]. When incorporating Chol up to 50 mol %, both fluid-like state bilayers maintain a homogeneous topography and a consequent unimodal *F_b_* distribution. In the case of the DOPC membranes, *F_b_* values remain approximately constant in the range of 10 and 17nN for low Chol contents, but increase up to around 29 nN for a Chol amount of 50 mol %. On the other hand, the mean *F_b_* values for the DLPC bilayers linearly increase with the Chol concentration ranging from 2 nN for the pure phospholipid to 8 nN for 50 mol % Chol [51]. The increase in *F_b_* values indicates an enhanced order and packing of the membrane, evidencing the condensing effect from Chol.

**Figure 4:**
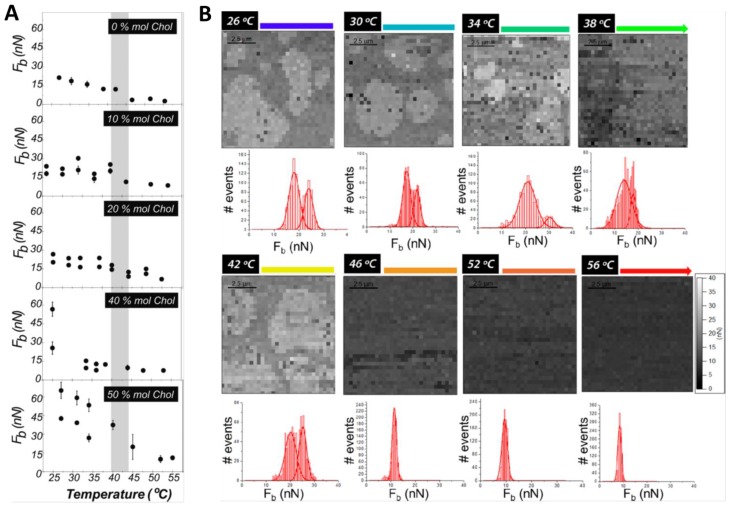
(**A**) Mean *F_b_* value of DPPC:Chol SPB in 10 mM HEPES, 20 mM MgCl_2_ and 150 mM NaCl, pH 7.4, with various Chol contents, as a function of temperature. The shadowed vertical line marks the temperature range where the main transition in pure DPPC occurs. For DPPC:Chol SPBs with 40 and 50 mol % Chol, although not detected in DSC of DPPC:Chol vesicles, a transition occurs around 42–45 °C; (**B**) *F_b_* maps and distributions for DPPC:Chol SPB with 10 mol % Chol, in 10 mM HEPES, 20 mM MgCl_2_ and 150 mM NaCl, pH 7.4, with increasing temperature. Adapted with permission from [9]. Copyright 2012 American Chemical Society.

At room temperature, DPPC forms gel phase SLB patches of about 5 nm height on mica surfaces, and when indented by AFM, it breaks with a mean *F_b_* value of about 22 nN [9,34]. When increasing the temperature, a slightly reduction of the *F_b_* value is observed until 45 °C, when the *F_b_*-temperature tendency clearly shows a break and mean *F_b_* values typical for fluid phase bilayers at room temperature (around 3.5 nN) are obtained (Figure 4A) [9,53]. It is evidenced that the mechanical stability of an SLB is highly dependent on the physical state of the lipid membrane. These observations are consistent with the DPPC thermal transition observed by differential scanning calorimetry (DSC), considering that the transition temperature (*T_m_*) of SLBs is usually slightly higher and broader than in liposomes suspension due to the influence of the underlying mica substrate [70]. In fact, structural changes can be observed during the transition range (42–50 °C), leading to the coexistence of different domains [69].

For gel-like state SLBs, the content of Chol is responsible for the behavior of the membrane, determining a homogeneous bilayer or separation into different domains. When low Chol contents, 10 and 20 mol %, are introduced in DPPC SLBs, two different phases coexist at room temperature (Figure 3A(a)), with a difference in thickness of about 300 pm. Consequently, AFM-FS measurements of these SLBs result in a bimodal *F_b_* distribution with two mean *F_b_* associated with each of the domains observed in the topography (Figure 3B). An *F_b_* value comparable to the one for pure DPPC bilayers (around 20 nN) is obtained for the lower and continuous phase, suggesting for this phase a low and constant Chol content. On the other hand, the second mean *F_b_* value increases with the overall Chol concentration (24 nN for 10 mol % and 27 nN for 20 mol %). This higher force value is associated with the higher domain observed in the topographical images, and can be defined as Chol-rich domains [9]. This correlation is exemplified in part A of Figure 3 for a DPPC:Chol SLB with 20 mol % Chol, where examples of typical force curves obtained for each domain are also shown. If the same experiment is performed under controlled increasing temperature, phase coexistence can be still observed until reaching 42–45 °C, with *F_b_* values that barely decrease during the heating (Figure 4B). With a further temperature increase, the bilayers become homogeneous and a corresponding unimodal *F_b_* distribution is obtained in the order of 10 nN. This corresponds to the homogenization and fluidization of the bilayers, since the systems have undergone the temperature range of the phase transition, in agreement with the broad transition observed with DSC [9]. Thus, the transition from a phase-segregated system to a homogeneous phase probably occurs gradually, with intermediate states that depend on the mobility and orientation of Chol within the membrane, as previously observed with quasielastic neutron scattering techniques [31].

Different behavior occurs when higher contents of Chol (higher than 30 mol %) are introduced into the DPPC bilayers, as most phase diagrams for the binary mixtures of DPPC:Chol suggest the existence of a unique liquid ordered (*l_o_*) state at any temperature for Chol compositions higher than 25–30 mol % [71,72,73,74]. AFM topographical characterization of DPPC:Chol SLBs at room temperature shows for 40 and 50 mol % Chol homogeneous membranes of about 3 nm height [9]. Although no microscopic domains are observed, when analyzed by AFM-FS these systems still show a bimodal *F_b_* distribution with extraordinary mechanical stability, displaying values almost three times higher than the one for the pure DPPC membrane (Figure 3B and Figure 4A) [9,51]. These bimodal distributions may be related to the presence of highly ordered small domains in dynamic equilibrium with less ordered lipid phases suggested by high spatial resolution neutron diffraction experiments on DPPC membranes containing 32 mol % Chol [75]. Upon heating the SLBs, a gradual decrease of the *F_b_* values is detected until reaching a temperature close to the physiological one (ca. 40 °C), where a unimodal distribution is observed with approximately constant values around 10 nN were determined for 40 and 50 mol % Chol (Figure 4A). Although the temperature/composition phase diagrams constructed for DPPC:Chol binary mixtures using DSC and ^2^H NMR propose the existence of a liquid ordered (*l_o_*) phase at all temperatures [71,76] and thermograms do not evidence any thermal transition for high Chol content vesicles, the decrease of the mean *F_b_* value indicates that the lateral molecular motion of the systems is increasing, meaning that a phase transition range is still present between 42 and 47 °C [9]. At higher temperatures, although the lateral mobility of these systems is still enhanced, they have higher lateral order compared to fluid phase DPPC bilayers. This suggests that a favorable structure with significant mechanical stability is obtained when equal amount of Chol and DPPC molecules are present in the bilayer, effect also observed in fluid-like state SLBs [9]. Moreover, volumetric measurements performed at temperatures above *T_m_* report that high Chol contents exhibit a relevant condensing effect on gel phase bilayers such as DPPC [77]. It then becomes clear that the influence of Chol on the bilayer ordering does not depend just on temperature, but is also associated with the state of the membrane.

## 4. Sphingolipids and Chol in Model SLBs

Biological membranes of eukaryotic cells contain large amounts of SLs together with Chol and the glycerophospholipids. In fact, it has been well established that nanoscale assemblies of lipids enriched in Chol, SLs, and proteins can be laterally segregated in the outer leaflet of the membrane [4,5]. These small domains are the so-called rafts, which are known to have an important influence on biological functions, such as membrane signaling and trafficking [4,6]. So, in addition to an extensive evaluation on how Chol affects the lipid membrane, it is important to consider the conjunct effect it plays together with SLs on the physical and nanomechanical properties of the lipid bilayer.

Sphingomyelin (SM) is the most prevalent membrane SL and is composed of a hydrophobic ceramide (Cer) moiety and a hydrophilic phosphocoline headgroup. When the hydrophilic group is a sugar, these are called glycosphingolipids (GSLs), like cerebrosides, when the sugar is glucose (glucosylceramide, GlcCer) or galactose (galactosylceramide, GalCer), or those with higher number of sugar moieties like globosides and gangliosides. They are all commonly found to be highly saturated in natural sources, and they are able to specifically modify the physical properties of the cell membranes [78]. Cer is one of the simplest SL found in cell membranes, also present in a significant fraction as an intermediate in the metabolism of more complex SLs. It is a major component of the stratum corneum preventing the evaporation of the water through the skin, due to its use as a hydrophobic barrier. Cer is found to have a significant role in cell signaling, since it is able to modulate the physical properties of biological membranes, leading to a reorganization of the membrane in response to stress signals [79]. Because of the high transition temperature and the extensive hydrogen bonding capability, Cer has a large impact on membrane properties, enhancing the ordering of the phospholipid molecules and producing lateral phase segregation as well as domain formation. In the case of SM, it is able to act as a hydrogen bond donor [80], although it does not display high transition temperatures compared to Cer or GalCer. GalCer are the major glycosphingolipids found in the central nervous system, primarily localized in the neuronal tissues [81,82], although GalCer are also significantly present in epithelial cells of the small intestine and colon, and in the granular sheath of the skin epidermis [83,84]. Also, because of the extensive hydrogen bonding capability of the saccharide headgroup, the *T_m_* of GalCer is particularly high (around 60 °C, depending on the composition), well above body temperature [80]. As a consequence, GalCer tend to be aligned in a compact manner, and involved in the formation of rafts in the outer leaflet of the membrane together with Chol [81,85].

### 4.1. Topography and Nanomechanical Stability by AFM

#### 4.1.1. Sphingomyelin

Several investigations have been performed on PC:SM:Chol systems due to the coexistence of both *l_o_* and *l_d_* phases mimicking lipid rafts. AFM and AFM-FS combined with fluorescence correlation spectroscopy (FCS) studies have shown a phase segregated SLB with a lower *l_d_* DOPC-rich phase, and higher domains in the *l_o_* state that are rich in SM and Chol, when the overall molar ratio DOPC:SM:Chol is 1:1:0.67 molar ratio [86]. By means of AFM-FS, the bilayer rupture of the *l_o_* domains in DOPC:SM:Chol occurs at *F_b_* around 10 nN, higher force value compared to the *l_d_* phase (around 6.5 nN) or to the pure DOPC bilayer (around 1.7 nN) [16,86], suggesting a higher degree of conformational order. In addition, the *l_o_* domains size increases with the increment of the Chol content from 10 to 35 mol %, until the *l_o_* phase becomes the matrix where the *l_d_* domains are dispersed, at 40 mol % Chol. Still, higher *F_b_* values always correspond to the SM- and Chol-rich *l_o_* domains, which range from 5.5 to 3.7 nN for Chol content of 15 to 25 mol %, respectively, while for the DOPC-rich *l_d_* phase, *F_b_* remains at 4–3 nN for such Chol concentrations [60]. A slight decrease in the nanomechanical stability of both coexisting phases, but more evidenced for the *l_o_* domains, was directly related to the increment of Chol content. A similar effect has been reported for DOPC:milk sphingomyelin (MSM) bilayers, where Chol not only affects the morphology of the MSM domains but also decreases their nanomechanical stability [87]. While DOPC:MSM (50:50 molar ratio) SLBs displayed *F_b_* of around 1.7 nN for the DOPC-rich continuous phase and 3–5.5 nN for the MSM-rich domains, upon 20 mol % Chol addition, the mean *F_b_* decreased to values lower than 1 nN.

AFM and AFM-FS have also been employed to characterize the active role of Chol in the physical properties of higher complexity mixtures like bilayer models of the milk fat globule membrane [88]. These membranes are principally composed of high *T_m_* polar lipids, mainly MSM that form domains in the gel phase or *l_o_* phase if mixed with Chol, and fluid-like matrix of unsaturated phospholipids (PE, PS, PI, and PC). Both in the continuous fluid phase and in the domains, the increase of the overall amount of Chol reduced the mechanical resistance, leading even to a homogenous fluid SBL for high Chol contents (beyond 27 mol %).

#### 4.1.2. Ceramide

**Figure 5:**
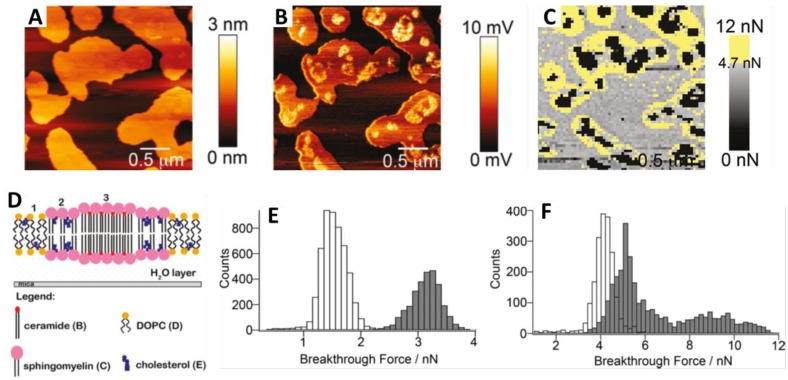
(**A**) AFM height image; (**B**) lateral deflection image; (**C**) the corresponding *F_b_* map; (**D**) illustration of phase segregated lipid bilayer with Cer-rich domains on mica; (**E**) *F_b_* histogram distribution of DOPC:SM:Chol (40:40:20 molar ratio) bilayer; (**F**) *F_b_* histogram distribution DOPC:SM:Chol:Cer (40:30:10:20 molar ratio) bilayer. Solid bars correspond to the *l_o_* domains, while hollow bars correspond to the *l_d_* phase. Adapted with permission from [91,92]. Copyright 2009 American Chemical Society.

As reported form AFM and FSC studies, DOPC:SM:Chol bilayers display three different topographical levels when a part of the SM content is replaced by Cer: a thinner *l_d_* phase enriched in DOPC, an intermediate *l_o_* phase enriched in SM and Chol, and a thicker one corresponding to domains rich in Cer together with SM [89,90]. These Cer-rich domains have an extremely high mechanical stability [91,92], confirming their tight lipid packing, most probably due to the strong affinity for hydrogen bonding with SM. In general, it has been determined that long-chain Cer incorporation leads to a lipid ordering and the whole mechanical stability of the membrane increases. It has been observed that Cer molecules could efficiently displace Chol from Chol:SM rich domains, increasing the presence of Chol in the DOPC-rich phase, reflected also in an increase of the *F_b_* [89,91,92,93]. While for SLBs of DOPC:SM:Chol (40:40:20 molar ratio) the mean *F_b_* values are around 1.4 nN for the *l_d_* and 3.2 nN for the *l_o_* phase (Figure 5E), when Cer (20 mol %) is incorporated (Figure 5A–D), these values raise to 4.1 and 5 nN, respectively, while the new Cer-rich domains were not able to be indented for the maximum forces applied in the reported experiments (Figure 5C,F) [91,92]. Still, short-chain Cer have been reported to modify the lipid packing decreasing the mechanical stability of lipid bilayers [6].

At the solubility limit of Chol, the addition of one more Cer molecules seems to displace Chol out of the bilayer, whereas Chol is not able to drive Cer out of the membrane [89,93,94]. Hence, the behaviors of Chol and Cer can be described with the so-called “umbrella model” [95], suggesting that both molecules compete for the coverage of PC headgroups to prevent the water contact of their nonpolar structures. Contrarily, it has been also latterly known that Chol increases the solubility of Cer in the fluid phase without depending on the presence of SM, indicating that both Cer and Chol have a complex portioning behavior. Therefore, the effect of Cer has a strong dependence on the concentration of Chol contained in the membrane, since at high Chol contents Cer seems to be solubilized in the fluid phase without gel phase formation [89], while at low Chol contents Cer and SM segregate in gel phase domains of high mechanical stability.

#### 4.1.3. Galactosylceramide

It has been determined that the domain formation in GalCer containing bilayers depends on the tail unsaturation of the PC lipid as well as on the content of Chol in the membrane. Although DPPC:GalCer SLBs with GalCer concentrations up to 20 mol % have been shown to display a homogenous topography by AFM, an increase in the mechanical stability has been reported with *F_b_* values from 11 nN for pure DPPC SLBs to 13 nN and 21 nN for 10 and 20 GalCer mol %, respectively [62]. For Chol contents lower than 8 mol %, coexistence of *l_d_* and solid ordered (*s_o_*) phases has been observed in (DOPC or POPC):GalCer:Chol systems [96], but after increasing the Chol content, the solid phase becomes *l_o_* and both liquid phases are present in the membrane. This behavior is similar to that observed with SM, although the transition to the *l_o_* phase is well established even before reaching the 8 mol % Chol. In the case of Cer, the *s_o_* domains remain solid-like still with concentrations of Chol higher than 20 mol % [97], as previously commented.

Phase segregated SLBs have been clearly visualized in DLPC:GalCer bilayers characterized by AFM, with GalCer being the main component of the higher domains, but also affecting the DLPC-rich region (lower continuous phase), leading to an increase in *F_b_*. From 2.7 nN for pure DLPC SLBs, 10 and 20 mol % GalCer lead to domains with an *F_b_* value around 42 nN, while the continuous DLPC-rich phase increases the mechanical stability to mean *F_b_* values of 8 and 15 nN for 10 and 20 GalCer mol %, respectively [62]. For the DLPC:GalCer:Chol system, the coexistence of both *l_d_* and *s_o_* phases remains up to 30 mol % [81]. For DLPC:Chol:GalCer (70:20:10 molar ratio), the SLB still shows two phases with mean *F_b_* values for each domain of 7 and 40 nN. Both phases display considerably higher nanomechanical stability than the DLPC:Chol (80:20 molar ratio) SLBs, although similar to DLPC:GalCer (90:10 molar ratio) SLBs. Hence, for low GalCer contents, 20 mol % Chol barely affects the SLB mechanical resistance [62].

Despite both GalCer and Cer showing *s_o_* domains, most probably due to the presence of intermolecular hydrogen bonds, the transition to a more liquid-like phase in the case of GalCer when working with high Chol contents can be associated with the larger headgroup compared to Cer. The behavior of the different phases is directly related to the strong interaction between Chol and the PC lipid molecules, noticing the preference of Chol for regions enriched with PC compared to ones rich in GalCer [81].

## 5. Forthcoming Steps: Coupling AFM with X-Ray Techniques

X-ray (XR) based techniques, such as reflectometry (XRR), grazing incidence small-angle XR scattering (GISAXS), and grazing incidence XR diffraction (GIXD), have been widely used to characterize the structural properties of biological surfaces at the nanoscale. XR has revealed many facts about the structural aspects of Chol in the lipid membrane. According to XR studies, the interaction of Chol is mainly determined by the chemical specificity of the lipid molecules [8]. In this way, it has been reported that Chol tends to compress saturated lipids by reducing their area, whereas lipids with unsaturated chains have weaker interactions with Chol, slightly screening such a significant condensing effect [13]. However, it has been determined that the lipid acyl chain length in mono-unsaturated SLBs has an essential impact on the orientation of Chol in the membrane [14]. Moreover, the lipid headgroups may rearrange the membrane organization when Chol is introduced (“umbrella model” [95]), minimizing the contact between the hydrophobic lipid chains and water.

Data are usually collected in synchrotrons, large-scale facilities providing XR beams with high brilliance. Synchrotron radiation permits us to investigate the structure of materials by providing the electronic density at high resolution. However, especially in grazing-incidence XR experiments, the information is usually averaged over the area illuminated by the beam footprint, which is covering a surface larger than that accessible by means of AFM. Therefore, a combination of XR with the local—nanometer scale—and mechanical information by AFM became powerful over the last decade [98,99,100,101,102,103]. So far, in situ correlative XR-AFM can give insights of dynamic processes, such as phase transitions or chemical reactions, as well as use the AFM tip to apply an external force or employ it to align a nano-object with the XR beam. In addition, AFM can also be used to evaluate the radiation damage induced by the XR beam in real time. Limiting radiation damage is a major challenge when using very intense XR beams on soft and biological samples. For instance, the formation of micrometric holes produced by an intense XR nanobeam on a semiconducting organic thin film has lately been observed in situ by means of HS-AFM [104].

**Figure 6:**
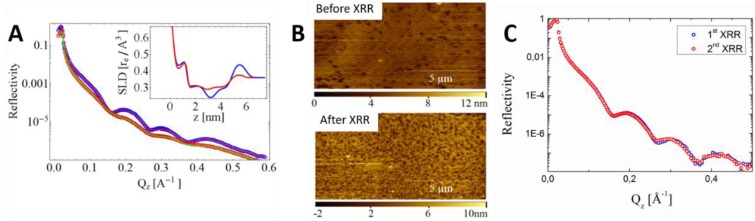
(**A**) XRR curves on DPPC bilayers. **Blue** and **red**: 1st XRR experimental data and best fit, respectively. **Red** and **green** (shifted for better clarity): 2nd XRR experimental data and best fit, respectively, acquired over the same sample region of the 1st XRR. Inset: SLD profiles evaluated from the fit. **Blue**: 1st XRR. **Red**: 2nd XRR; (**B**) AFM images of DPPC bilayers: (**left**) before being exposed to XR, (**right**) after being damaged by the XR beam during the acquisition of the 1st XRR (22.5 keV). Adapted with permission from [105]; (**C**) XRR curves on DPPC bilayers. **Blue**: 1st XRR experimental data. **Red**: 2nd XRR experimental data, acquired over the same sample region of the 1st XRR (30 keV); Comparing (**A**) and (**C**), it is evidenced that 30 keV produces less radiation damage to the SLBs.

In all the previously referenced cases, some of the mechanical elements of the AFM limited the applications to the field of material science, preventing the possibility of exploring biological samples under liquid environment, such as SLBs. Recently, a fast AFM has been developed and successfully tested in a synchrotron beamline, extending the capabilities to biological applications [105]. In particular, simple DOPC and DPPC SLBs were first studied using the XR-AFM setup, which allowed us to evaluate radiation damage. Radiation damage was observed on these SLBs under liquid conditions, determining, from both AFM and XR data, a decrease of the membrane coverage produced by the exposure of the XR beam (22.5 keV) (Figure 6A,B). While the scattering length density (SLD) profiles obtained from the XRR data (Figure 6A-inset) clearly show an averaged decrease of the membrane coverage, the AFM image collected after XR exposure (Figure 6B) additionally shows the nanometric size of the holes formed in the membrane. Minimizing radiation damage is one of the key issues to reinforce the use of XR over neutron techniques, with higher resolution and faster measurements, to study biological-related films [106]. Accordingly, we have recently discovered that when increasing the XR energy to 30 keV no radiation damage on phospholipid SLBs is evidenced. This novel approach allowed us to acquire two consecutive XRR datasets in the very same sample region of DPPC SLB (Figure 6C), without radiation damage effects.

**Figure 7:**
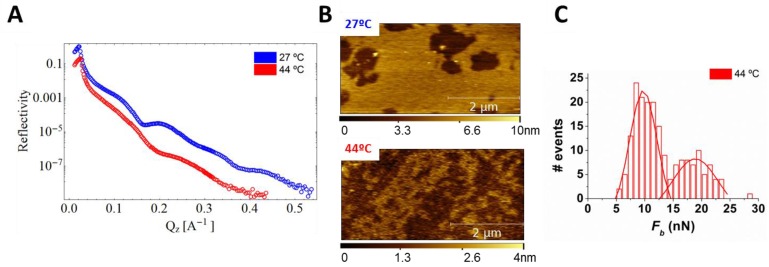
(**A**) XRR curves on DPPC bilayers at 27 °C (**blue**) and 44 °C (**red**); (**B**) AFM topographical images at 27 °C (**top**) and 44 °C (**bottom**); (**C**) *F_b_* histogram distribution for the DPPC SLB at 44 °C.

Moreover, the combined XR-AFM setup permits in situ characterization of dynamic processes such as phase transitions, providing structural, morphological, and mechanical information. Temperature-induced phase transition of DPPC membranes occurring at approximately 44 °C clearly shows membrane thinning, highlighted by the increase of the oscillation periods in XRR data compared to XRR data at room temperature (Figure 7A blue and red curves, respectively). This is likely occurring because of an increase in phospholipid disorder at 44 °C. Comparison of AFM images collected below and above the *T_m_* (Figure 7B) shows membrane remodeling from DPPC patches with an average thickness of 3.5 nm to coexistence of domains of different thickness (0.5 nm difference in thickness between them) that we interpret as DPPC gel and liquid phases. In addition, the local information provided by AFM permits us to characterize the size of the domains, ranging from a few tens to hundreds of nm^2^. The simultaneous presence of two membrane phases is supported by the mechanical information collected by means of AFM-FS: the *F_b_* distribution measured in the very same region of the AFM image at 44 °C (Figure 7C) clearly shows a bimodal distribution with higher *F_b_* for gel phase compared to fluid phase. As a consequence, our data suggest that the DPPC fluid phase is less ordered (XRR) and this directly affects the interaction between lipid molecules diminishing *F_b_*.

The large amount of data that can be collected at once in a single correlative XR-AFM experiment permits us to fully characterize membrane dynamic transitions, providing structural and morphological information from nanoscale (XRR) to the mesoscale (AFM) as well as complementary mechanical insights.

Since the XR-AFM setup for biological applications is a recent development, only results concerning simple SLBs have been obtained so far. However, we are convinced that in situ correlative XR-AFM can give new insight into the structure–mechanics relationship in complex bilayers, including Chol and SLs, and will allow the evaluation of not only the chemical composition and structural effect on mechanical stability but also the effects of mechanical force on the structure and reorganization.

## 6. Concluding Remarks

Despite the high mechanochemical complexity of biological membranes, simplified models like SLBs have been shown to be good platforms to evaluate the lipid membrane physical properties and the contribution of different components like Chol and SLs to their morphological and mechanical stability. To this end, AFM and AFM-FS have become crucial experimental techniques, due to the possibility of locating and probing confined areas of membranes at the nanometer scale, under controlled environmental conditions and with nano- to piconewton sensitivity.

Chol plays an important role in adjusting the physical properties of biological membranes, managing the membrane fluidity and mechanical resistance, by controlling the organization and phase behavior of the lipid bilayer. While Chol has been shown to phase segregate in gel-like SLBs when the content is low, and when higher than 30 mol % Chol leads to a homogeneous SLB both in fluid and gel phase SLBs, AFM-FS has proved that it enhances the mechanical stability in all cases. Temperature-controlled AFM-FS has been able to detect a thermal transition for high Chol content SLBs, even when the temperature/composition classical phase diagrams for DPPC:Chol mixtures propose the existence of an *l_o_* phase at all temperatures. Topographical and nanomechanical characterization by AFM has shown how Chol is involved in the membrane reorganization when coexisting with different SLs (SM, Cer, and GalCer), directly affecting the domains and lipid distribution, modulating their mechanical stability.

We finally introduced the great potential of the combination of AFM techniques with those based on XR to allow the study of dynamic processes providing in situ structural, morphological, and nanomechanical information—for instance, the effect of small molecules’ and peptides’ interaction with the lipid membrane on its physical properties. This combination will, for instance, allow us to follow the effect of composition on the membrane structure, but also the result of applying an external force on compositional changes and the restructuring of the membrane.
